# Antineutrophil Cytoplasmic Antibody-Associated Vasculitis With Acute Kidney Injury: Short-Term Recovery Predicts Long-Term Outcome

**DOI:** 10.3389/fimmu.2021.641655

**Published:** 2021-07-09

**Authors:** Xiaohan Huang, Liangliang Chen, Lan Lan, Pingping Ren, Anqi Ni, Yanhong Ma, Yaomin Wang, Yilin Zhu, Ying Xu, Jianghua Chen, Fei Han

**Affiliations:** ^1^ Kidney Disease Center, The First Affiliated Hospital, Zhejiang University School of Medicine, Hangzhou, China; ^2^ Institute of Nephrology, Zhejiang University, Hangzhou, China; ^3^ Key Laboratory of Kidney Disease Prevention and Control Technology, Zhejiang Province, Hangzhou, China

**Keywords:** ANCA associated vasculitis, acute kidney injury, end stage renal disease, mortality, outcome

## Abstract

**Background:**

Kidney involvement is common in antineutrophil cytoplasmic antibody (ANCA) associated vasculitis (AAV). It tends to be aggressive, and in some patients, the kidney involvement may reach the criteria of acute kidney injury (AKI). Here, we aim to describe the clinical characteristics of these patients and find risk factors for poor outcomes.

**Methods:**

Patients diagnosed with AAV in our hospital from February 2003 to February 2017 were included. Those who reached the KDIGO AKI criteria were reclassified according to the KDIGO AKI stage. The clinical features of these patients were analyzed. Also, according to the variation of serum creatinine 3 months after AKI episode, patients were further divided into two groups: patients whose serum creatinine (Scr) level at the third month decreased by 30% or more from the peak Scr level was classified into G1 and others were classified into G2. Long-term renal and survival outcomes of these patients were analyzed with a Cox model. The renal endpoint was reaching end-stage renal disease (ESRD), and the survival endpoint was death. Nomograms were built based on cox models.

**Results:**

Of 141 AAV patients included, during the median follow-up period of 64.0 (IQR 34.8, 85.4) months, 36 (25.5%) patients reached renal endpoints, and 22 (15.6%) patients died. The median renal survival time was 35.9 (IQR 21.3, 72.6) months and the median survival time was 48.4 (IQR 26.8, 82.8) months. Multivariate analysis showed that poor recovery of Scr level at 90 days (P < 0.001, RR = 9.150, 95%CI 4.163–20.113), BVAS score (P = 0.014, RR = 1.110, 95% CI1.021–1.207), and AKI stage 3 (P = 0.012 RR = 3.116, 95%CI 1.278–7.598) were independent risk factors for renal endpoints; poor recovery of Scr level at 90 days (P = 0.010, RR = 3.264, 95%CI 1.326–8.035), BVAS score (P = 0.010, RR = 1.171, 95%CI 1.038–1.320) and age (P = 0.017, RR = 1.046, 95%CI 1.008–1.086) were independent risk factors for all-cause death. The c-index of nomograms is 0.830 for the renal outcome and 0.763 for the survival outcome.

**Conclusion:**

KDIGO AKI stage 3 is the risk factor for ESRD in AAV patients with AKI. The BVAS score and level of kidney function recovery at 90 days are the independent risk factors for both ESRD and all-cause death and are of predictive value for the outcome.

## Introduction

Kidney involvement is common in antineutrophil cytoplasmic antibody (ANCA) associated vasculitis (AAV) (1, 2). It occurs in about 90% of patients with microscopic polyangiitis (MPA), and about 70% of patients with granulomatosis with polyangiitis (GPA) (3). A typical manifestation of renal involvement is aggressive kidney vasculitis (4). It can present with a rapid decline of kidney function and may need renal replacement therapy within a few days, and even lead to end-stage renal disease (ESRD). In some patients, the clinical course of kidney injury can be very aggressive and can reach the criteria of acute kidney injury (AKI).

AKI is characterized as sudden renal impairment. Studies have shown that AKI is a risk factor for ESRD, and is associated with poor survival outcomes (5). The criteria and staging system proposed by the Kidney Disease: Improving Global Outcome (KDIGO) group based on the time and level of increase of creatinine or decrease of urine output is recommended for evaluating the severity of AKI and predicting renal outcomes (6, 7).

There are limited approaches to evaluate the level of progression of renal injuries caused by AAV. Here we retrospectively analyzed AAV patients who reached the KDIGO AKI criteria in our center, trying to describe their characteristics, as well as finding out the associations between AKI stage or short-term kidney function recovery and long-term outcomes.

## Methods

### Patients

**Figure 1:**
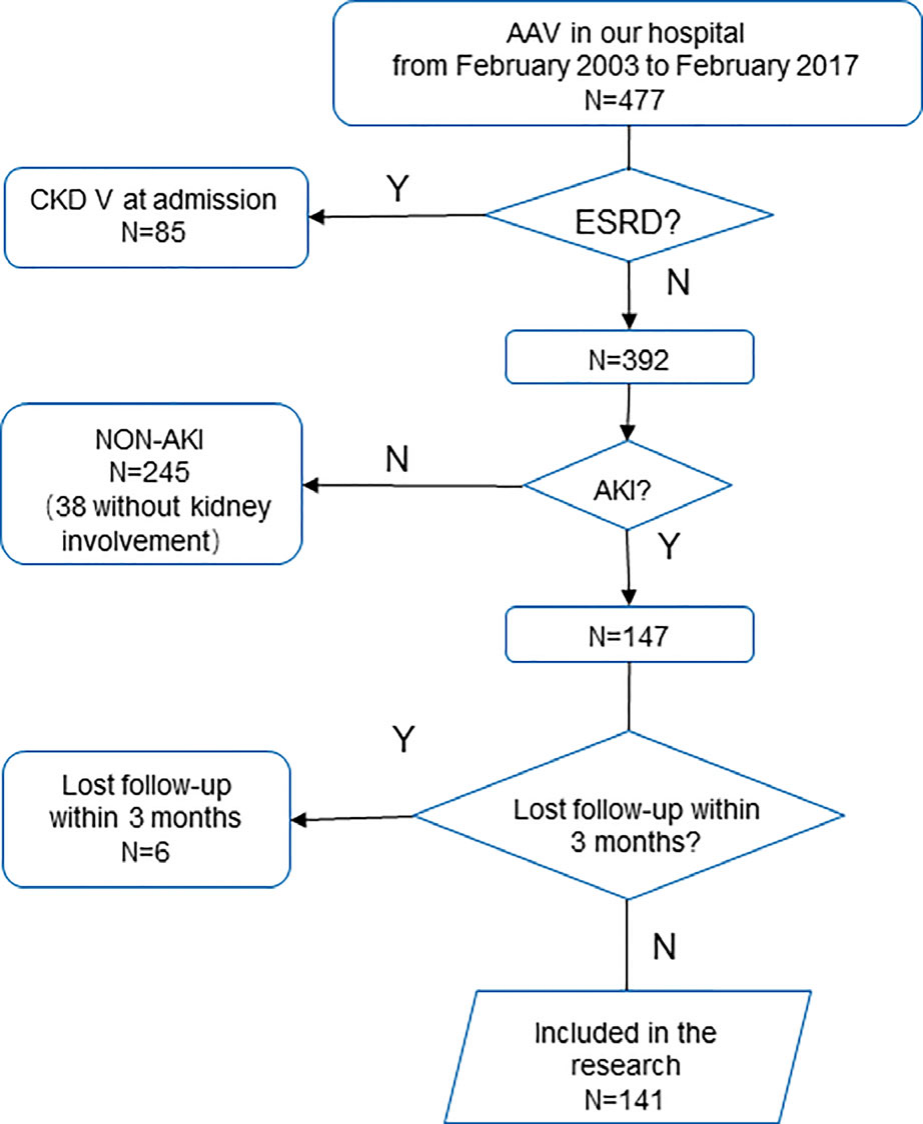
Flowchart.

Patients diagnosed with AAV in our hospital from February 2003 to February 2017 according to the Chapel Hill consensus (1) were retrieved. Those who were diagnosed with ESRD at admission were excluded. Patients who reached the KDIGO criteria for AKI, and followed up for more than 3 months or reached the primary endpoint within 3 months were included (**Figure 1**). Based on the KDIGO guideline, AKI is defined as serum creatinine (Scr) increased more than 0.3 mg/dl (26.5 μmol/l) within 48 h, Scr raised to a 1.5-fold baseline within 7 days, or a decreased urine output less than 0.5 ml/kg/h over 6 h (8). The research protocol was approved by the Research Ethics Committee of the First Affiliated Hospital, Zhejiang University School of Medicine.

Generally, patients with ANCA-associated vasculitis were treated with corticosteroids (0.6–0.8mg/kg/d) with intravenous cyclophosphamide (CTX) 15 mg/kg every 2–4 weeks until the cumulative dose reached 6–8 g; or corticosteroids with mycophenolate mofetil (MMF) 1.0–1.5 g/d (9–11). Patients with pulmonary hemorrhage, biopsy-proven cellular crescents, or fibrinoid necrosis of small vessels were treated with reinforcement therapy of 500 mg pulse methylprednisolone for 3 days before CTX or MMF therapy. Renal replacement therapy was adopted as supportive treatment when patients were with severe hyperkalemia or metabolic acidosis, signs of uremia, or refractory fluid overload.

### Data Collection

Clinical data including age, gender, blood pressure, smoke, medical history, organs involved, urine output, urine protein, urine red blood cell, the level of C-reactive protein (CRP), myeloperoxidase (MPO)-ANCA or proteinase 3 (PR3)-ANCA, therapies used, and renal biopsy report were retrieved through medical records. Patients were staged according to the KDIGO clinical practice guidelines for AKI (8). And if there were two or more recorded AKI episodes, only the first episode was analyzed. All patients were evaluated with the Birmingham Vasculitis Activity Score (BVAS) version 3 according to their status at admission. Vascular damage index (VDI) at admission is also evaluated (12). Survival data were acquired from digital records of regular follow-up visits, telephone follow-ups, and the dialysis database of Zhejiang Province.

The kidney functions of patients were evaluated through Scr levels. Scr level on the day of admission, peak level within 7 days (in the AKI episode), the level at the third month’s follow-up, and the level at last follow-up were recorded. Baseline Scr was acquired through previous records or the level at admission (pre-episode). For patients who do not have previous records, the baseline Scr was back-calculated through the MDRD equation assuming an estimated glomerular filtration rate (eGFR) of 75 ml/min/1.73m^2^ (6, 13).

According to the change of Scr level, patients with AKI were further divided into two groups. Patients whose Scr level at the third month decreased by 30% or more from the peak Scr level was classified into subgroup 1 (G1), and others were classified into subgroup 2 (G2). The cut-off point is defined by the approximation of the lower tertile.

### Outcome Definition

As chronic kidney diseases are defined as loss of kidney function or markers of kidney damage for more than 3 months (14), the endpoint of many AKI studies was 60- or 90-day mortality (15). In this study, short-term outcomes were evaluated by the status of patients 3 months after the episode. The renal endpoint was ESRD, and the survival endpoint was defined as death from any cause. The composite endpoint was defined as reaching renal endpoint or survival endpoint. ESRD was defined as kidney failure that reaches an estimated glomerular filtration rate (eGFR) lower than 15 ml/min/1.72 m^2^ or needs maintaining renal replacement therapy for more than 3 months.

### Statistical Analysis

For continuous data, normally distributed data were expressed as average ± deviation, while non-normally distributed data were reported with median (interquartile range). Counting data was expressed with frequency, rate, or proportion. Student-t test, one-way ANOVA, Student–Newman–Keuls, Wilcoxon, and Kruskal–Wallis tests were used to compare continuous data. Counting data were compared with cross-table or Fisher’s exact test.

Survival analysis was conducted with the Kaplan–Meier survival analysis, log-rank test, logistic regression, and Cox proportional hazards model. Factors in the univariate Cox model with P-value <0.1 were further included in multivariate analysis, and the forward LR model was used in the multivariate model. P <0.05 was considered significant.

The multivariate Cox regression model was used to build the final nomogram prognostic model, the accuracy of the model was assessed with discrimination and calibration (16, 17). The concordance index (c-index, a generalization of the area under the ROC curve) was used to assess the discrimination power of the model, and graphical plots showing observed outcomes and predicted probabilities are used for calibration.

The statistical analysis was done by SPSS 23.0 (IBM, Chicago, IL, USA). The prognostic model and its assessment were conducted in the R environment, version 3.5.3, and R packages “survival”, “rms” were used.

## Results

### Demographic and Clinical Features

In 477 patients retrieved, 85 patients diagnosed with ESRD at admission were excluded. Approximately 147 patients reached the AKI criteria, while 245 did not.

In 147 patients with AKI, six patients lost to follow up within 90 days were further excluded. Finally, 141 patients were included. In these patients, 65 (46.1%) were male, and the median age was 59.6 (51.4, 67.5) years old. The lung is the most commonly involved extrarenal organ, followed by skin and joints (**Table S1**, in **Supplementary Material**).

Patients were staged according to the KDIGO guidelines. There are 43 patients in AKI stage 1, 44 patients in AKI stage 2, and 54 patients in AKI stage 3 (Additionally, the number and percentage of patients defined by urine output or serum creatinine were listed in **Table S2**). As shown in **Table 1**, patients in AKI stages 1 to 3 had different BVAS at admission (P = 0.038). The median and interquartile ranges were 16.1 ± 3.8, 15.3 ± 3.5, and 17.3 ± 3.9 respectively. Also, the levels of CRP, serum albumin, and Scr are statistically different (P <0.001). There are no significant differences in other clinical and laboratory characteristics.

**Table 1:** Clinical and laboratory data of AAV patients in different AKI stages.

	Stage 1 (n = 43)	Stage 2 (n = 44)	Stage 3 (n = 54)	P-value
Age, Median (IQR)	59.7(53.4,64.9)	58.2(42.0,66.9)	60.3(52.6,69.8)	0.155
Male, n (%)	22(51.2)	21(47.7)	22(40.7)	0.573
Smoke, n (%)	11(25.6)	14(31.8)	12(22.2)	0.842
Hypertension, n (%)	21(48.8)	22(50.0)	24(44.4)	0.558
Scr at admission, μmol/l, Median (IQR)	171.0(121.0,270.0)	224.5(161.0,309.8)	308.0(232.3,509.5) ^**##^	**<0.001**
Baseline Scr, μmol/l, Median (IQR)	116(90,188)	125(82.5,156.75)	132.0 (83.3,184.0)	0.484
Urine red blood cell /ul, Median (IQR)	188.4(86.8,505.5)	429.9(93.0,1331.7)	406.1(80.3,1225.0)	0.162
White blood cell ×10^9^/L, Median (IQR)	7.0(5.1,10.2)	7.7(5.6,10.0)	8.6(6.5,10.3)	0.130
Hemoglobin g/L, Median (IQR)	87.0(77.0,100.0)	87.0(79.0,99.0)	80.0(71.0,93.5)	0.079
Platelet×10^9^/L, Median (IQR)	265.0(187.0,338.0)	202.5(158.0,285.5)	226.0(166.75,289.5)	0.074
24h urine protein g, Median (IQR)	1.56(0.72,3.02)	1.75(1.13,3.47)	1.95(0.79,2.75)	0.434
Albumin g/L, Median (IQR)	33.9(29.9,37.8)	34.1(31.3,37.8)	28.6(25.6,31.1) ^**##^	**<0.001**
Globulin g/L, Median (IQR)	29.4(26.3,34.3)	30.7(24.5,34.3)	30.0(26.8,32.9)	0.973
CRP mg/L, Median (IQR)	14.6 (4.2, 58.9)	10.5 (3.3, 58.1)	40.4 (18.8, 85.8) ^**##^	**<0.001**
MPO-ANCA positive, n (%)	29(67.4%)	33(75.0%)	34(62.9%)	0.443
MPO, Median (IQR)	50.0(22.7,78.4)	66.0(19.4,100.0)	40.5(12.9,150.0)	0.928
PR3-ANCA positive, n (%)	4 (9.3%)	7 (15.9%)	5 (9.3%)	0.516
PR3, Median (IQR)	1.4(1.1,2.0)	1.5(1.13,2.68)	1.4(1.1,2.9)	0.823
BVAS, Mean ±std.	16.1±3.8	15.3±3.5	17.3±3.9^#^	**0.038**
VDI at admission, Median (IQR)	0 (0,1)	0 (0, 1)	0 (0,1)	0.699
Treatment				
I.V. Pulse Methylprednisolone	26(60.5%)	24(54.3%)	34(63.0%)	0.752
Immunosuppressants, n (%)				
CTX	19 (48.8%)	21 (47.7%)	28 (51.9%)	0.752
MMF	21 (48.8%)	18 (40.9%)	19 (35.2%)	0.309

Scr, serum creatinine; MPO, myeloperoxidase; BVAS, Birmingham Vasculitis Activity Score; VDI, vascular damage index. **P < 0.01, compared with AKI stage1; #P < 0.05, compared with AKI stage 2; ##P < 0.01, compared with AKI stage 2.

Bolded value: P-value < 0.05.

Renal biopsy was performed in 96 (68.1%) patients, and the pathological characteristics are presented in **Table 2**. The proportion of glomeruli with crescents was 11.9% (5.7%, 34.1%) in stage 1 patients, 16.1% (10.9%, 33.4%) in stage 2 patients, and 21.2% (14.3%, 51.7%) in stage 3 patients (P = 0.043), showing an upward tendency. Differences in other pathological parameters did not reach statistical significance (P >0.05).

**Table 2:** Pathological characteristics of AAV patients in different AKI stages.

	Stage 1 (n = 32)	Stage 2 (n = 31)	Stage 3 (n = 33)	P-value
Global sclerosis^1^, Median (IQR)	20.3 (10.0, 38.5)	21.1 (12.5, 36.4)	15.4 (7.1, 20.8)	0.510
Crescent^1^, Median (IQR)	11.9 (5.7, 34.1)	16.1 (10.9, 33.4)	21.2 (14.3, 51.7) ^**^	**0.043**
Cellular crescent^1^, Median (IQR)	5.8 (0.0, 21.4)	8.0 (2.0, 13.4)	15.2 (5.7, 26.7)	0.075
Capillary Necrosis, n (%)	12 (37.5)	11 (35.4)	19 (57.5)	0.140
Mesangial hypercellularity, n (%)	32 (100.0)	31 (100.0)	32 (96.970)	0.381
Endocapillary hypercellularity, n (%)	9 (28.1)	13 (41.9)	16 (48.5)	0.232
Interstitial fibrosis, n (%)				0.399
0–50%	26 (81.2)	22 (71.0)	22 (66.7)	
>50%	6 (18.8)	9 (29.0)	11 (33.3)	
Interstitial infiltration, n (%)				0.269
0–25%	11 (34.4)	4 (12.9)	8 (24.2)	
25–50%	11 (34.4)	11 (35.5)	9 (27.3)	
>50%	10 (31.2)	16 (51.6)	16 (48.5)	

^1^Proportion of glomeruli per biopsy with the lesion, data was expressed as median and interquartile range (IQR).

**P < 0.01, compared with AKI stage 1.

Bolded value: P-value < 0.05.

### Short-Term Renal Recovery and Outcomes

After 3 months, patients were divided into subgroups (G1 and G2) according to the variation of Scr level. There are 90 patients in the G1 subgroup and 51 patients in the G2 subgroup.

In the cohort, 84 (59.6%) patients received i.v. pulse methylprednisolone therapy, 68 (48.2%) patients used cyclophosphamide, and 58 (41.1%) patients used mycophenolate mofetil. There are no significant differences in treatment between the two groups (**Table 3**).

**Table 3:** Clinical and laboratory data of AAV patients in G1 and G2 subgroups.

	G1 (n = 90)	G2 (n = 51)	P-value
Age, Median (IQR)	60.3 (52.7, 66.7)	58.6 (51.0, 70.2)	0.597
Male, n (%)	37 (41.1)	28 (54.9)	0.114
Smoke, n (%)	23 (25.6)	14 (27.5)	0.806
Hypertension, n (%)	42 (46.7)	25 (49.0)	0.340
Scr at admission, μmol/l, Median (IQR)	257.0 (162.0, 384.5)	209.0 (157.5, 287.0)	0.075
Baseline Scr, μmol/l, Median (IQR)	117.0 (81.2, 155.5)	134.0 (99.0, 190.0)	0.070
Urine red blood cell/ul, Median (IQR)	406.1 (134.3, 1302.9)	139.3 (77.9, 631.2)	**0.006**
White blood cell × 10^9^/L, Median (IQR)	7.6 (5.4, 10.0)	8.5 (6.6, 10.4)	0.063
Hemoglobin g/L, Median (IQR)	83.0 (76.2, 95.0)	82.0 (71.5, 101.5)	0.555
Platelet×10^9^/L, Median (IQR)	217.0 (167.8, 291.8)	244.0 (182.0, 303.5)	0.298
24 h urine protein g/L, Median (IQR)	1.7 (1.0, 2.7)	1.9 (0.7, 3.3)	0.324
Albumin g/L, Median (IQR)	32.6 (28.1, 36.7)	31.0 (27.1, 35.0)	0.284
Globulin g/L, Median (IQR)	30.5 (27.0, 33.2)	29.5 (25.9, 35.0)	0.804
CRP mg/L, Median (IQR)	21.5 (6.6, 58.4)	33.1 (4.6, 101.7)	0.109
MPO-ANCA positive, n (%)	67(74.4)	37 (68.5)	0.806
MPO, Median (IQR)	53.1 (20.3, 116.0)	42.7 (17.9, 94.8)	0.138
PR3-ANCA positive, n (%)	10 (11.1%)	6 (11.8%)	0.906
PR3, Median (IQR)	1.5 (1.1, 2.8)	1.3 (1.1, 2.0)	0.382
BVAS, Mean ± std.	16.6 ± 3.7	15.8 ± 4.2	0.175
VDI at admission, Median (IQR)	0 (0, 1)	0 (0, 1)	0.117
AKI stage, n (%)			0.154
Stage 1	24 (26.7)	19 (37.3)	
Stage 2	33 (36.7)	11 (21.6)	
Stage 3	33 (36.7)	21 (41.2)	
Treatments, n (%)			
I.V. Pulse Methylprednisolone	58 (64.4)	26 (51.0)	0.117
Plasma exchange	3 (3.3)	2 (3.9)	0.856
Immunosuppressants, n (%)			
CTX	42 (46.7)	26 (51.0)	0.622
MMF	42 (40.0)	16 (27.5)	0.093
Renal biopsy, n (%)	64 (71.1)	32 (62.7)	0.496
Status at 3-month, n (%)			
Withdrew dialysis	14 (15.6)	5 (9.8)	**<0.001**
Dialysis	0 (0)	18 (35.3)	
Death	0 (0)	2 (2.1)	
Long-term outcome			
Death, n (%)	8 (8.9)	14 (27.5)	**0.007**
Survival time, month, Median (IQR)	56.5 (27.5, 82.0)	41.0 (24.5, 86.0)	0.480
Renal endpoint, n (%)	12 (13.3)	24 (47.1)	**<0.001**
Renal survival time, month, Median (IQR)	48.0 (26.2, 80.8)	24.0 (3.0, 39.0)	**<0.001**
Composite endpoint, n (%)	16 (17.8)	28 (55.0)	**<0.001**

Scr, serum creatinine; MPO, myeloperoxidase; BVAS, Birmingham Vasculitis Activity Score; CTX, cyclophosphamide; MMF, mycophenolate mofetil; VDI, vascular damage index.

Bolded value: P-value < 0.05.

At the time of discharge, 14 patients in the G1 subgroup were still with renal replacement therapy, and all of them successfully withdrew dialysis at the 3rd month; 23 patients in the G2 subgroup were with renal replacement therapy at the time of discharge, and five (21.7%) of them withdrew dialysis at the third month. Two patients in the G2 subgroup died within 3 months without recovery of kidney function. There were no significant differences in clinical or laboratory characteristics between the two subgroups except for urine red blood cells [406.1 (134.3, 1302.9)/ul *vs* 139.3 (77.9, 631.2))/ul, P = 0.006] (**Table 3**). As for pathological characteristics, 64 (71.1%) patients in G1 and 32 (62.7%) patients in G2 had received renal biopsy (P = 0.496), and pathological parameters of the two groups showed no significant difference (**Table S2** in the **Supplementary Material**). During long-term follow up, 16 (17.8%) patients in the G1 subgroup and 28 (55.0%) patients in the G2 subgroup reached composite endpoint (p <0.001) (**Table 3**).

### Long-Term Outcomes and Predictive Model

During a median follow-up of 64.0 (34.8, 85.4) months, 36 (25.5%) patients reached renal endpoint and 22 (15.6%) patients died. The median renal survival time was 35.9 (21.3, 72.6) months, and the median overall survival was 48.4 (26.8, 82.8) months.

**Figure 2:**
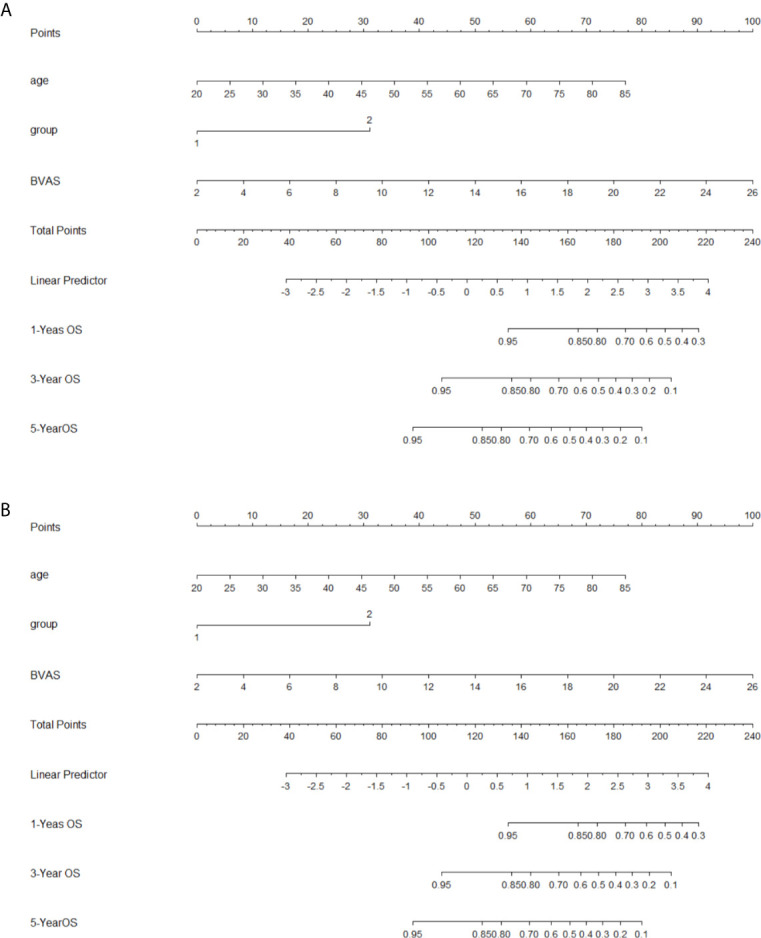
Nomograms for outcomes **(A)**, nomogram for renal outcome; **(B)**, nomogram for survival outcome; To use the nomogram, based on patient’s value, draw upward lines from each variable axis to the points, and calculate the sum of these points. And draw a downward line from total point axis to the survival axis to get the likelihood of 1-, 3-, 5-year survival).

In the multivariate model (**Table 4**), after adjusted for age and gender, AKI stage 3 (P = 0.012 RR = 3.116, 95%CI 1.278–7.598), high BVAS score (P = 0.014, RR = 1.110, 95% CI1.021–1.207) and G2 subgroup (P <0.001, RR = 9.150, 95%CI 4.163–20.113) were independent risk factors for poor long-term kidney outcome in AAV patients with AKI. These factors (AKI stage, subgroup, BVAS) were used to construct the nomogram for predicting the renal outcome (**Figure 2A**) (calibration plot in the **Supplementary Material**), by internal validation the c-index was 0.830.

**Table 4:** Cox model for long-term outcome.

Factors	Univariate model for renal survival			Multivariate model for renal survival			Univariate model for overall survival			Multivariate model for overall survival		
	RR	95%CI	P-value	RR	95%CI	P-value	RR	95%CI	P-value	RR	95%CI	P-value
G(G2)	6.156	2.920-12.977	**<0.001**	9.150	4.163-20.113	**<0.001**	3.286	1.378-7.835	0.007	3.264	1.326-8.035	**0.010**
BVAS	1.094	1.004-1.192	0.041	1.110	1.021-1.207	**0.014**	1.152	1.029-1.289	0.015	1.171	1.038-1.320	**0.010**
AKI1			0.057	/	/	0.038	/	/	0.101			
AKI2	0.909	0.347-2.380	0.846	1.711	0.639-4.582	0.286	0.932	0.249-3.489	0.916			
AKI3	2.116	0.923-4.951	0.077	3.116	1.278-7.598	**0.012**	2.434	0.791-7.490	0.121			
Age	1.007	0.980-1.035	0.604				1.062	1.021-1.104	0.003	1.046	1.008-1.086	**0.017**
Male	1.510	0.779-2.928	0.222				1.307	0.563-3.035	0.533			
24h urine protein	1.188	0.984-1.433	0.073				1.209	0.962-1.520	0.104			、
Urine red blood cell	1.116	0.573-2.173	0.747				1.324	0.554-3.165	0.528			

BVAS, Birmingham Vasculitis Activity Score; AKI, acute kidney injury. Forward LR method was adapted in multivariate models.

Bolded value: P-value < 0.05.

As for long-term survival outcome, age (P = 0.017, RR = 1.046, 95%CI 1.008–1.086), BVAS (P = 0.010, RR = 1.171, 95%CI 1.038–1.320), and G2 subgroup (P = 0.010, RR = 3.264, 95%CI 1.326–8.035) were found to be the risk factors (**Table 4**). The risk factors (age, subgroup, BVAS) were used to construct the nomogram for predicting the renal outcome (**Figure 2B**, calibration plot in the **Supplementary Material**), by internal validation the c-index was 0.763.

## Discussion

Kidney involvement is common in patients with AAV. Although treatments for AAV have improved in the past decades, about 20–25% of patients progress into ESRD several years after diagnosis and need persistent dialysis or kidney transplantation (18, 19). Our study suggests that in AAV patients with AKI, both BVAS at admission and the level of kidney function recovery in three months can be independent predictors for ESRD and mortality, and the KDIGO AKI stage is a predictor for the poor renal outcome. The result provided potential indicators for both early identification of high-risk patients and prediction of long-term prognosis.

AKI stage is defined as the proportional rise of Scr and is classified according to the severity of kidney injury, and the severity of the renal injury is suggested to be related to long-term outcomes (20). In AAV patients, the KDIGO AKI stage also shows renal outcome predictive value. Of note, in the research, the AKI stage is not a predictor of long-term survival outcomes. One hypothesis is that AKI does not directly impact the mortality in AAV patients. Some researchers suggested that the intervention of AKI may not have a significant impact on mortality. For example, in situations including post-operation with low mortality risk or sepsis, though AKI is associated with higher mortality, it is not sure to what degree of AKI works as a marker for unrecognized risk of mortality (15).

In this study, several features showed significant differences among different AKI groups. BVAS score and CRP level were significantly higher in AKI stage 3 patients, suggesting that acute exacerbation was related to disease activity and inflammation. Though without statistically significant difference in pairwise comparison, the mean BVAS score of AKI stage 1 was higher than stage 2. The BVAS score shows systemic active injury, however, the activity of injury may vary in different tissues or organs. Further analysis found that pulmonary hemorrhage was more frequently seen in stage 1 patients (supporting material). So, the activity in organs outside the kidney may contribute to the relatively high BVAS score. Decreased albumin level was also found in AKI stage 3. Generally, hypoalbuminemia can be caused by increased urine loss or increased consumption during inflammation or chronic disease. We showed that there was no difference in urine protein level among groups, while a higher CRP level was found in group AKI stage 3. We found that in these patients, serum albumin level was correlated with CRP level (**Supplementary Material**). So, the hyperinflammatory response may contribute to hypoalbuminemia. Previous studies have also found that high inflammation marker levels were related to lower serum albumin, and it is suggested that inflammation suppresses albumin synthesis (21). The presentation of crescent formation and tubular infiltration increased with the AKI stage. The proportion of cellular crescents showed a trend of increase but was not of statistical significance, and we infer that the significance of differences is limited by sample size. Previously, some studies have found correlations between histological features and patient outcomes. Former histopathologic studies classified ANCA-associated glomerulonephritis into four types: focal, crescentic, mixed, and sclerotic (22), the sclerotic type has the poorest outcome, followed by the crescentic type (23). Brix et al. developed a renal risk score system based on tubular atrophy, interstitial fibrosis, the proportion of normal glomeruli, and eGFR at diagnosis (24). Another study found that the renal risk score which combines histologic and laboratory features performs better in predicting the renal outcome (25).

BVAS at admission were independent risk factors of ESRD or mortality. It is used to evaluate disease activity while the Vasculitis Damage Index (VDI) evaluates long-term organ damage (12). Our finding consists of previous studies that disease activity is associated with outcomes (26).

Besides the AKI stage and BVAS at diagnosis, the recovery of kidney function at 3 months showed the potential of predicting renal and survival outcomes. This indicates assessment at 3 months may also provide important information about prognosis. In clinical studies of AKI, kidney recovery is an important short-term (60–90 d) endpoint, and if kidney damage lasts for 3 months, it and can be classified into CKD. In AAV, Gopaluni et al. indicated that disease activity 3 months after diagnosis may predict long-term outcomes (26). Our study showed that after acute exacerbation of kidney function, early follow-ups are also helpful for clinical management. Comparing to a single measurement of serum creatinine, the fold change of creatine level may better reflect the dynamic process of post-AKI recovery or damage. A single measurement of Scr cannot reflect the real-time kidney function, and the level of serum creatinine can be influenced by acute diseases, muscle mass. Some researchers presumed that there is a new baseline in patients recovered from AKI that lower than the original one, and recovery within a certain range may suggest a sustaining renal injury (15). Though how short or medium-term outcomes can be translated into long-term outcomes is not fully understood, our research indicated that the recovery of serum creatinine at 3 months can be a predictive factor of long-term outcomes.

Interestingly, the level of urine red blood cells at admission is significantly higher in G1 than in G2. We performed univariate and multivariate models, but the level of urine red blood cells did not show association with renal (P = 0.747, RR = 1.116, 95%CI 0.573–2.173) or survival outcome ((P = 0.528, RR = 1.324, 95%CI 0.554–3.165). The reason for this phenomenon is not clear. Furthermore, in the cohort, the level of urine red blood cell is not associated with long-term outcomes. Knowledge about the role of hematuria in ANCA-associated vasculitis is limited and more studies are needed (27).

There some limitations to this study. This study is a single-center retrospective study, and the number of patients with GPA or EGPA is limited. Also, in this retrospective cohort, BVAS score and VDI score ex-renal activity and damages at follow-ups of more than 50% of patients were irretrievable, so only renal outcomes and survival outcomes of patients are analyzed. We also acknowledge that due to the limitation of retrospective study, treatment differences between individuals exist. This may mask the relationship between some clinical features and long-term outcomes. However, on the whole, there were no significant differences in therapies used among different AKI stages or between different groups (**Tables 1**, **3**). We hope our conclusion can be validated in prospective multi-center cohorts.

## Conclusion

KDIGO AKI stage3 is the risk factor for ESRD in AAV patients with AKI, while the BVAS score and level of kidney function recovery at 90 days are the independent risk factors for ESRD and all-cause mortality, and is of predictive value.

## Data Availability Statement

The raw data supporting the conclusions of this article will be made available by the authors, without undue reservation.

## Ethics Statement

The studies involving human participants were reviewed and approved by Research Ethics Committee of the First Affiliated Hospital, Zhejiang University School of Medicine. The patients/participants provided their written informed consent to participate in this study.

## Author Contributions

XH contributed to the conception of the study, analysis, and manuscript preparation, performed the data analyses, and wrote the manuscript. LC contributed to resource provision, maintenance of data, and performed the data analyses. LL and PR contributed to resource provision and maintenance of data. AN, YM, YW, and YZ helped perform the analysis with constructive discussions. JC and FH contributed to commentary and revision of the manuscript. FH also contributed to funding acquisition. All authors contributed to the article and approved the submitted version.

## Funding

This study was supported by the funds from the Primary Research and Development Plan of Zhejiang Province (2020C03034) to FH, Zhejiang Medical and Health Science and Technology Project (2018258985, 2019RC036) to LC and LL, and Project of Natural Science Foundation of Zhejiang Province (Q19H050030) to PR.

## Conflict of Interest

The authors declare that the research was conducted in the absence of any commercial or financial relationships that could be construed as a potential conflict of interest.
