# Supplementary material for: Antineutrophil Cytoplasmic Antibody-Associated Vasculitis With Acute Kidney Injury: Short-Term Recovery Predicts Long-Term Outcome
Source: Front Immunol. 2021 Jul 9;12:641655. doi: 10.3389/fimmu.2021.641655 (PMC8299708; doi:10.3389/fimmu.2021.641655)
Supplement: Supplementary file 1 [file Table_1.docx]

# Tables

# Table S1. General information of AAV patients

| Items | AKI (n=147) | Non-AKI (n=245) |
| --- | --- | --- |
| Age, Median (IQR) | 60.0(51.0, 68.0) | 65.0(56.0,73.0) |
| Male, n (%) | 66(44.9) | 101(41.2) |
| Smoke, n (%) | 37(25.2) | 53(21.6) |
| AKI stage, n (%) |  |  |
| Stage 1 | 43(30.5) | / |
| Stage 2 | 44(31.2) | / |
| Stage 3 | 54(38.3) | / |
| Extrarenal organ involvement, n (%) |  |  |
| Lung | 46(31.3) | 64(26.1) |
| Joints | 22(15.0) | 17(7.0) ** |
| Ear-Nose-Throat | 10 (7.1) | 19(8.1) |
| Skin | 20(13.6) | 18(7.4) * |
| Eye | 5(3.4) | 9(3.7) |
| Central nervous system | 6(4.1) | 19(7.8) |
| Abdomen | 16(11.0) | 27(11.1) |
| Scr at admission, mol/l, Median (IQR) | 274.0(163.0, 420.5) | 156(77.5, 371.0) ** |
| BVAS, mean±std. | 16.3±4.0 | 13.7±5.5** |

*:P<0.05; **:P<0.01

**Table S2. Distribution of AKI stage and outcomes defined by urine output or serum creatinine**

|  | Patient number, n (%) | Renal endpoints n (%) | Death, n (%) |
| --- | --- | --- | --- |
| Defined by urine output | 22 (100%) | 9 (40.9%) | 5 (22.7%) |
| AKI Stage 1 | 3 (13.6%) | 1 (33.3 %) | 0 (0%) |
| AKI Stage 2 | 3 (13.6%) | 1 (33.3 %) | 0 (0%) |
| AKI Stage 3 | 16(72.7%) | 7 (43.8%) | 5 (31.2%) |
| Defined by serum creatinine change | 119 (100%) | 27 (22.7%) | 17 (14.3%) |
| AKI Stage 1 | 40 (33.6%) | 7 (17.5%) | 4 (10.0%) |
| AKI Stage 2 | 41 (34.5%) | 8 (19.5%) | 5 (12.2%) |
| AKI Stage 3 | 38 (31.9%) | 12 (31.6%) | 8 (21.1%) |

AKI, acute kidney injury.

**Table S3. Pathological characteristics of G1 and G2 subgroups**

|  | G1(n=64) | G2(n=32) | P-value |
| --- | --- | --- | --- |
| Global sclerosis^1^, Median (IQR) | 16.7(9.1,34.7) | 23.2(11.9,38.5) | 0.265 |
| Crescent^1^, Median (IQR) | 16.1(10.0,36.1) | 18.9(9.4，39.6) | 0.456 |
| Cellular crescent^1^, Median (IQR) | 9.7(0.0,19.5) | 9.2(3.2,21.9) | 0.402 |
| Necrosis, n (%) | 28 (43.8) | 14 (43.8) | 0.934 |
| Mesangial proliferation, n (%) | 64 (100.0) | 31 (96.9) | 0.302 |
| Endocapillary hypercellularity, n (%) | 23 (35.9) | 15 (46.9) | 0.302 |
| Tubular infiltration, n (%) | 27 (42.2) | 19 (59.4) | 0.112 |
| Interstitial fibrosis, n (%) |  |  | 0.230 |
| 0-50% | 44 (68.8) | 26 (81.2) |  |
| ＞50% | 20 (31.2) | 6 (18.8) |  |
| Interstitial infiltration, n (%) |  |  | 0.727 |
| 0-20% | 17 (26.6) | 6 (18.8) |  |
| 20-50% | 19 (29.7) | 11 (34.4) |  |
| ＞50% | 28 (43.8) | 15 (46.9) |  |

**Table S4. Number and percentage of patients with pulmonary hemorrhage**

| Massive hemoptysis / alveolar hemorrhage | AKI stage 1 | AKI stage 2 | AKI stage 3 | P-Value |
| --- | --- | --- | --- | --- |
| Yes | 14 (32.6%) | 5 (11.4%) | 9 (16.7%) | 0.035 |
| No | 29 (67.4%) | 39 (88.6%) | 45 (83.3%) |  |

# 2.Caliberations

Calibrations for renal outcome model

(1-,3-5-year)


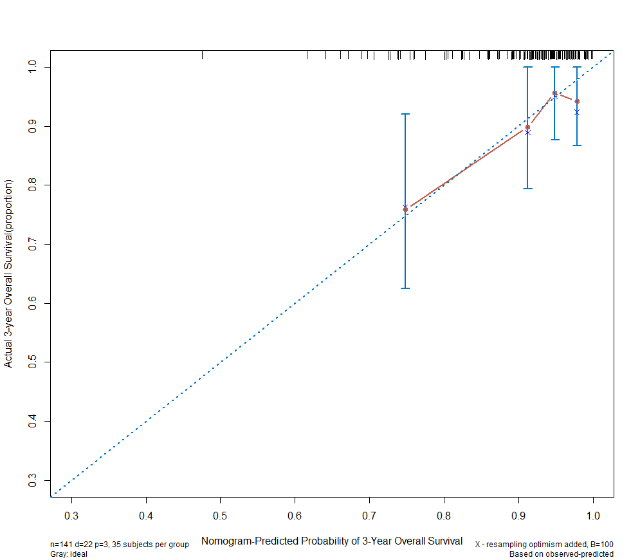

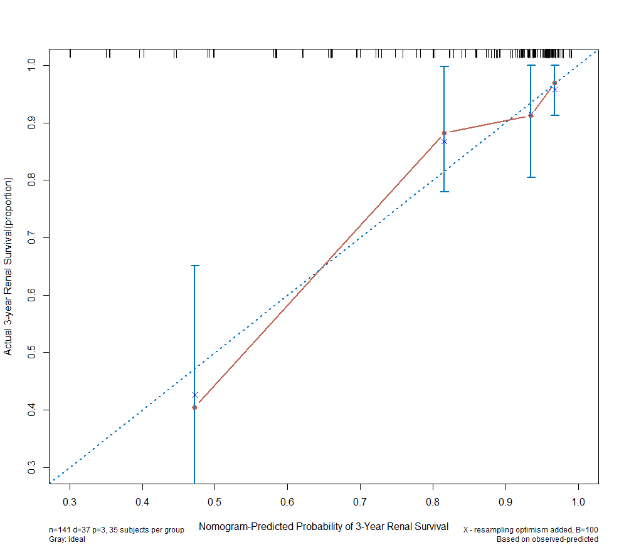

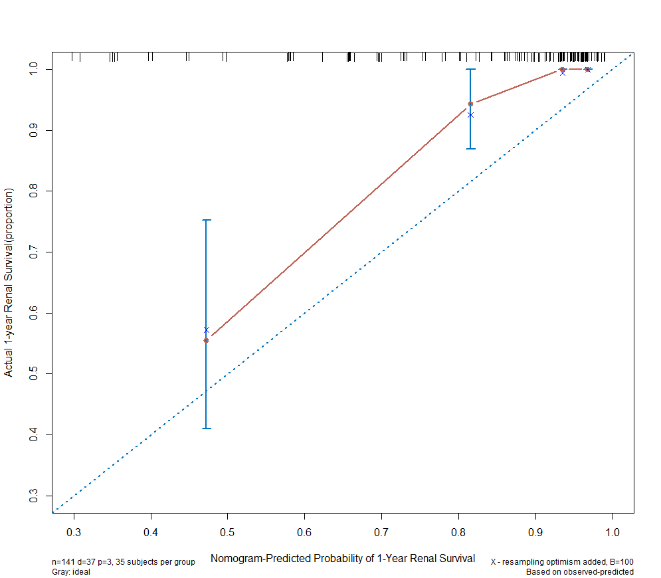

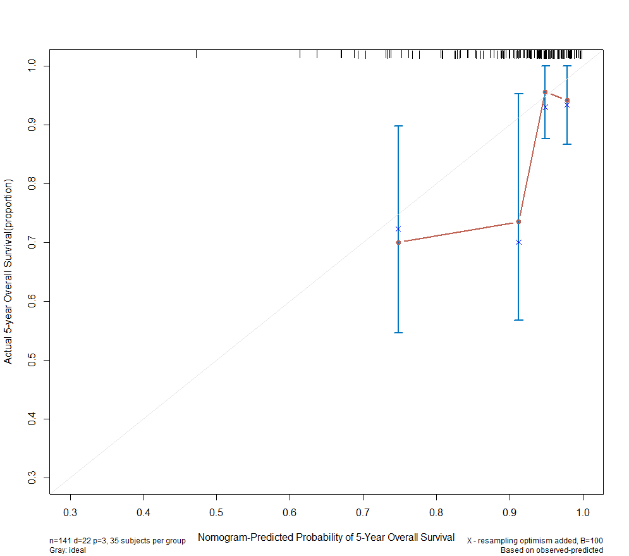


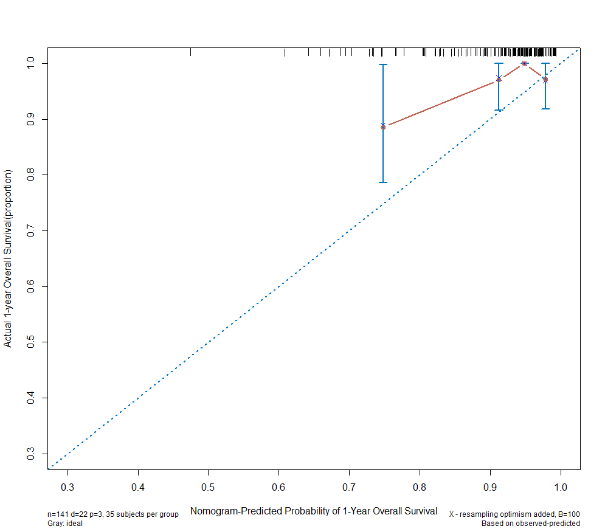
Calibrations for renal outcome model

(1-,3-5-year)


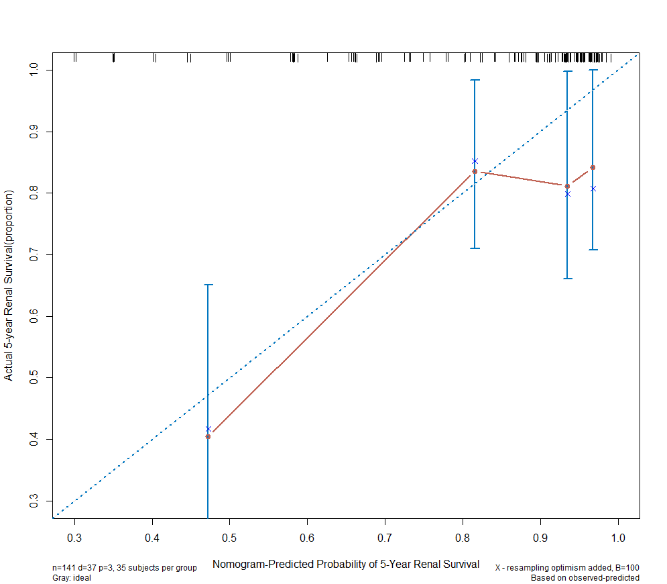


# 3. CRP& Serum albumin


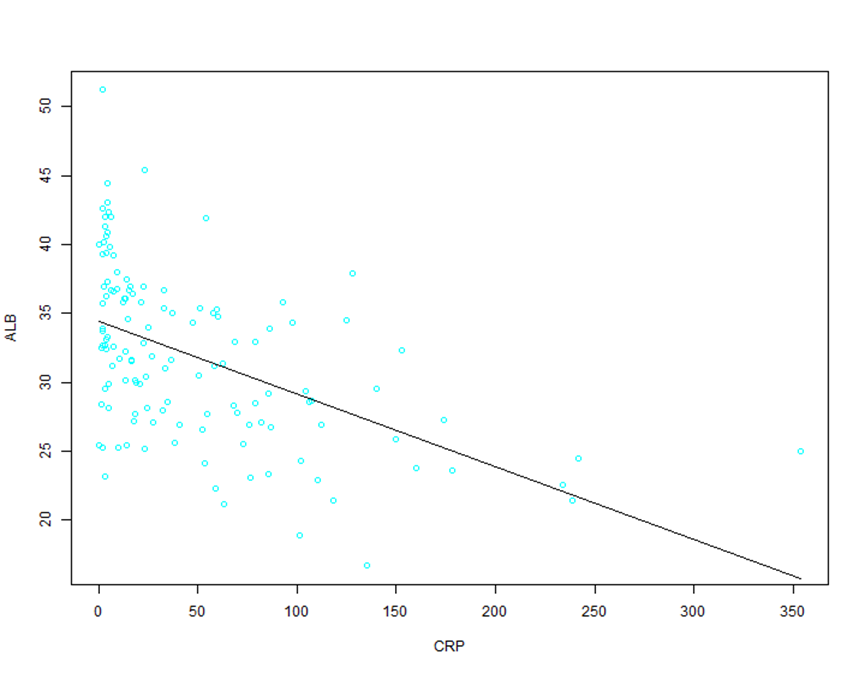


R=-0.537, P<0.001
